# Racially Equitable Homeless Services: Exploring Organizational Characteristics

**DOI:** 10.1177/2752535X251321535

**Published:** 2025-02-20

**Authors:** Whitney Thurman, Elizabeth Heitkemper, Tara Hutson, Summer Wright, Amy Patten, Andrea Kaltz

**Affiliations:** 1 12330 The University of Texas at Austin, Austin, TX, USA; 2 Sunrise Homeless Navigation Center, Austin, TX, USA; 3 Homeless Strategy Division, City of Austin, Austin, TX, USA

**Keywords:** homelessness, racial equity, qualitative, community-engaged methods

## Abstract

**Purpose:**

Racial disparities in homelessness are pervasive and necessitate sustained effort on improving racial equity in homeless services. This study used a community-engaged approach and qualitative methods to describe the role of informal organizations identified by Black adults with lived experience of homelessness as preferred locations for accessing services and to explore the values and beliefs of these informal organizations. The study included representatives (*N =* 19) of community organizations (*N* = 17) in one southern city. Most participants (*n* = 14, 73.6%) worked in paid positions and included executive directors as well as volunteers.

**Findings:**

Thematic analysis identified three themes that characterized values and the ways in which these organizations interface with one another and with the formal homelessness response system (HRS): *boots-on-the-ground, the homelessness response system is inequitable,* and *cautious collaboration.* Findings reveal avenues through which local collaboration can be improved and potential policies to improve racial equity in homeless services.

**Conclusions:**

Informal organizations fill critical gaps in services and can reach people experiencing homelessness who are unable or unwilling to access formal services. However, informal organizations often remain disconnected from the larger HRS which can exacerbate racial inequities. Community care hubs are a promising solution to incorporating smaller organizations and building a more integrated and equitable HRS.

## Introduction

Racial disparities in homelessness are stark and well-documented.^1^ In 2023, 37% of people experiencing homelessness (PEH) in the United States self-identified as Black or African American despite representing only 13% of the general American population (National Alliance to End Homelessness, 2023).^2^ These racial disparities are evident across the homelessness response system as research has identified that Black individuals who exit homelessness to permanent housing return to homelessness more frequently and more quickly than do white individuals.^3^ Further, Black adults often experience differing pathways into initial homelessness^4^ resulting in differential service use patterns, needs, and barriers to successfully exit homelessness.^5,6^ Unfortunately, little research has explored the characteristics of the organizations that Black adults experiencing homelessness identify as preferred locations for accessing services. The present study uses a community-engaged approach and qualitative methods to identify and assess key characteristics, values, and beliefs of a sub-set of community-based organizations providing services to PEH. We focus specifically on organizations that are not a formal part of the homelessness response system or continuum of care.

### The Homelessness Response System

#### Continuums of Care

In response to the persistent issue of homelessness across the U.S., an array of social services, health care, and housing supports are provided by public, private, and nonprofit entities to support PEH. Such networks of stakeholders are typically knit together into continuums of care (CoC) in which efforts are coordinated to meet health and social needs and support PEH to exit homelessness. CoCs were initiated in 1994 by the U.S. Department of Housing and Urban Development (HUD) in response to the need for cross-sector collaboration to improve the outcomes of PEH. In 2009, HUD established the CoC Program as the primary route of providing funding for housing and homelessness. The CoC is also responsible for community-level planning to meet the needs of PEH.

In recognition of the pervasive racial disparities in homelessness, HUD has incentivized local communities to prioritize racial equity in their responses to homelessness by providing greater funding opportunity for CoCs actively working to reduce racial disparities (HUD, 2018, FR-6200-N-25).^7^ Subsequently, CoCs have pursued racial equity at the local level through several routes. Recent evidence has identified that CoCs focus on procedural fairness and representation through actions such as adopting anti-discrimination policies; forming diversity, equity, and inclusion (DEI) committees and policies; and reserving seats on the CoC governance board for Black, Indigenous, people of color (BIPOC) in order to be representative of local communities (Kim et al., 2023).^8^ Other efforts center on implementing data-driven decision-making processes that can improve equity (Cronley et al., 2024).^9^ This research has also identified equity in service provision as a barrier facing CoCs that contributes to inefficiencies and creates service gaps within local communities (Mosley, 2021).^10^ That is, federal funding often dictates target populations for CoCs and leaves little room for local communities to adjust program criteria to equitably meet local need.^11^

#### Homeless Services

Health and social services are crucial components of homelessness response. The extent to which an individual experiencing homelessness accesses needed services is influenced by several factors. These include the availability of information and awareness of services, transportation barriers, and strict eligibility criteria requirements for many federally-funded services.^5^ Prior evidence also suggests that frequency of service use is not always predicted by level of need^12^ and that a substantial percentage of PEH do not engage with homeless services at all.^13,14^ Research has also documented higher rates of homeless services use among Black as compared to white PEH,^15–17^ but the limited research investigating the effectiveness of those services for Black individuals has demonstrated mixed results in terms of homelessness exit and housing outcomes.^18^

Homelessness is deeply stigmatized,^19,20^ and homelessness stigma has been identified as a significant deterrent to accessing services contributing to an array of poor outcomes.^5^ In fact, stigma within healthcare settings has been associated with greater length of homelessness.^21^ As members of a minoritized racial group, Black individuals experiencing homelessness carry the additional burden of race-based discrimination on top of homelessness stigma. Research has identified that Black PEH report higher levels of discrimination than do white PEH,^22^ and perceived racial stigma and discrimination within service settings has been found to lead to service avoidance by Black PEH and to be independently associated with poor physical health.^23^ Prior evidence also suggests that homeless services are often structured in ways that reinforce deficit-oriented views^24^ and perpetuate negative racial stereotypes.^25^ In order to minimize these experiences of racial discrimination and stigmatization, research has found that Black adults often seek services and support outside of the formal homelessness response system.^3^ Taken together, the current evidence base suggests a critical need to understand characteristics of the array of service providers that are available in a community with particular focus on those that may be preferred by Black PEH.

## Current Study

Rectifying racial disparities necessitates attention to the structural injustices that place minoritized racial groups at risk of experiencing homelessness in the first place, but the homelessness sector must also critically examine the ways in which systems may reproduce and reinforce racial disparities. A critical aspect of equity is individual choice and preference which is not always accounted for within CoC decision-making or homeless service settings. We currently lack empirical evidence that clearly describes organizations identified by Black PEH as preferred providers of homeless services that are not part of the CoC (henceforth referred to as informal organizations). Evidence regarding how these organizations operate and whether there are disparities in efficacy or access is an important step towards ensuring that racially equitable and culturally safe homeless services are integrated across the homelessness response system in local communities. The purpose of the current study, therefore, was to first describe the role of informal organizations identified by Black PEH as providing health and/or social services in culturally safe and acceptable ways. We then explored the values and beliefs of those organizations and examined how they interface with one another and with the formal homelessness response system (HRS) and CoC.

### Sensitizing Framework

Fundamental cause theory^26^ was the overarching theoretical framework. According to this theory, stigmatized individuals and populations have less access to health-promoting resources such as money, knowledge, power, and beneficial social connections. Subsequently, individuals with stigmatized social identities have less ability to avoid health risks and to minimize the sequelae of illness once it occurs.^26^ Social network theory facilitated a structural understanding of the community by identifying how individuals, institutions, and organizations are linked as well as by illuminating how social networks operate and how resources flow within them.^27,28^ These concepts provided points of departure for our data collection and served as a foundation for the interpretation and analysis of data^29,30^

### Research Design & Methods

Guided by principles of community-based participatory research (CBPR)^31^ this study extended efforts of the lead agency for the local continuum of care (CoC) to explore accessibility of the HRS for Black PEH. In 2022, the local CoC conducted an asset-mapping project to quantify local resources with a particular focus on identifying those organizations and entities that were not part of the formal HRS. That project also explored the perspectives of Black adults with lived experience of homelessness regarding barriers to accessing services in the local area. The current study extended that work by exploring characteristics of a subset of organizations identified in the asset-mapping project. Because the original project focused on organizations outside of the HRS, we likewise focused on informal organizations. We operationalized informal as any entity that provides services or support to PEH but does not receive specific governmental funding (local, state, or federal) to provide those services.

We addressed our research questions through a community-engaged study using a qualitative descriptive design in which representatives from informal organizations participated in interviews, focus groups, or both. Qualitative description is well-suited for exploratory research such as the current study because it facilitates detailed description and descriptive validity through the examination of text that represents participants’ experiences and perspectives.^32–34^ suggests that the qualitative toolbox offers multiple methods to enhance the exploration of complex phenomena and goes further to encourage researchers to carefully employ multiple qualitative methods within a single study if needed to enhance the analysis and broaden the conceptualization of the phenomena under study.^33,34^ Thus, we used both individual interviews and focus groups to gain a more comprehensive understanding of the role of informal organization in providing homeless services. Interviews focused on organizational values and structure, services provided, how those services are delivered, and how individual values align with organizational values before probing for additional insights related to cross-sectoral collaboration via focus groups. Interview and focus group guides are found in the online supplementary material. This study was guided by the Consolidated Criteria for Reporting Qualitative Research (COREQ) guidelines.^35^ It was deemed exempt by the University Institutional Review Board.

#### Key Informants

The lead author of this manuscript collaborated with the lead agency of the CoC to recruit individuals who had participated in the asset-mapping project to serve as key informants for this study. Seven ultimately accepted the invitation and participated as key informants (*n* = 6 male; *n =* 1 LGBTQ identified). These individuals were consulted about their experiences of navigating the community to access services and attaining resources necessary for survival and for exiting homelessness. The first (W.T) or third (T.H.) author met with these individuals from one to three times to both confirm names of organizations from which they felt comfortable accessing services as well as reasons for such. As the study progressed, we modified our interview guide and added organizations to our sampling frame based on key informant feedback. Key informant input served as a method of triangulation, and we also checked emerging interpretations of our data with key informants. Key informants received $45.00 cash at the end of each meeting.

#### Sample, Setting, and Recruitment

Individuals were eligible to participate in this study if they were ≥18 years and were involved with an organization identified by a key informant. Participation was open to anyone who was employed by or volunteered with an identified organization and was not limited to a specific organizational role. Upon identification, the research team sent an informational email to an organizational representative providing the study’s purpose and expectations. The email included a link to a Qualtrics survey that contained relevant information and an option to assent to participate. Upon the participant’s assent, the survey proceeded with demographic questions and scheduling of interviews. For those who did not assent, the survey ended by thanking them for their time. Interviews occurred between July and November 2022, and all were completed before conducting the focus groups in November and December 2022. Participants received $50.00 cash in appreciation of their time and expertise.

#### Data Collection

Interviews were conducted remotely using Zoom or in-person, depending upon the participant’s preference. All focus groups were conducted remotely on Zoom. The first author of this study (W.T.) independently conducted 15 interviews, and the third author (T.H.) independently conducted four. Each interview lasted approximately 1 hour. The first author facilitated all three focus groups/group interviews with the second, third, or fourth authors in attendance for note-keeping. It should be noted that while we intended to conduct focus groups with 4-5 participants each, scheduling difficulties resulted in only one focus group (*n* = 5 participants) supplemented with a dyadic interview and a three-person interview. Focus groups/group interviews lasted between 1.25 and 1.5 hours. Audio recordings of each interview and focus group were professionally transcribed and de-identified upon verification.

#### Data Analysis

Thematic analysis was facilitated through NVivo, a qualitative data management program. The goal of the analysis was to construct a thematic description^36^ of key elements delineating participants’ perceptions of the role of informal organizations within the local HRS, to explore the values and beliefs that guide the work of informal organizations, and to identify the extent to which informal organizations collaborate with one another and with the formal HRS. Our analysis had interrelated inductive and theoretical components. Specifically, the coding process was inductive and linked to the data without a pre-existing coding frame so that themes could emerge independent of the research team’s prior knowledge and experience.^37^ However, the conceptualization of themes was influenced by both fundamental cause and social network theories.

The first four authors met for two initial coding sessions during which this interdisciplinary research team collaboratively segmented and labeled two focus group transcripts to generate an initial coding schema. Patterns of meaning in the data were examined and discussed during regular team meetings ultimately resulting in an initial code book. Next, we identified themes based on explicit meanings in the data. For example, data coded as *“ability to innovate”* and *“client access”* was collaboratively reviewed and ultimately collapsed into the category *“services & support.”* At this point, we reviewed the coding structure to refine and organize the final set of themes and categories which were agreed upon by the project team. The interview transcripts and remaining focus group transcripts were divided amongst the team to code independently. We met as a group two more times to reach consensus on thematic saturation.^38^

#### Rigor, Validity, and Reliability

Procedures for credibility, transferability, dependability, and confirmability were incorporated throughout this research to ensure trustworthiness. First, the research team includes two non-academic members who work in homeless services in the community in which this study was conducted and were involved in all aspects of the study. Ongoing reflexive dialogue among the research team ensured attention to assumptions that might influence the study and our analysis. We also reviewed our guides and emerging interpretations of the data with our key informants. The use of NVivo software allowed us to ensure that findings are representative of the data as it tracked the frequency of codes, linked them to the data, and provided an audit trail. Other procedures included taking field notes, team debriefing, reflexive journaling, consideration of negative cases. Finally, rich description of the study context and participant quotes allows readers to gauge transferability.^39^

## Results

### Sample Demographics

Table 1 presents the participants’ demographic characteristics. The final sample included representatives (*N =* 19) of informal organizations (*N* = 17) that were identified by our key informants as providing essential services to PEH and that met our definition of informal. Over half (*n* = 10, 52.6%) reported lived experience of homelessness. Participants represented diverse organizational perspectives and job titles ranged from executive director to volunteer. Most participants (*n* = 14, 73.6%) worked in paid positions. Table 2 presents characteristics that were identified by participants as salient to their organization missions and services. We confirmed services and year of founding via organizational websites.

**Table 1.** Demographic Characteristics of Participants (*N* = 19).

Participant characteristics	
Gender identity, *n* (%)^a^	
Female	11 (57.9)
Male	6 (31.6)
Transgender	2 (10.5)
Non-binary/non-conforming	1 (5.2)
Prefer not to respond	1 (5.2)
Race/Ethnicity, *n* (%)	
Black/African American	7 (36.8)
White	7 (36.8)
Hispanic/Latiné	2 (9.5)
Multiethnic	2 (9.5)
Other	1 (5.2)
Lived experience of homelessness, *n* (%)	
Yes	10 (52.6)
No	9 (47.4)
Length of time involved with homeless services, *n* (%)	
Less than 1 year	1 (5.2)
1-3 years	5 (26.3)
4-6 years	3 (15.8)
7-10 years	6 (31.6)
Longer than 10 years	4 (21.1)
Organizational role, *n* (%)	
Executive director	6 (31.2)
Program director^b^	5 (26.3)
Volunteer, not further specified	4 (21.1)
Pastor	2 (10.5)
Care coordinator^c^	2 (10.5)

^a^*n* does not equal 19 due to participants claiming multiple gender identities.

^b^Includes: organizing director, food pantry director, clinical services director, volunteer coordinator, shelter director.

^c^One individual served in a care coordinator position on a volunteer basis.

**Table 2.** Organizational Characteristics.

	Characteristics	501c3 status (Y/N)	Services provided	Year founded
#1	Black-led	Yes	Transitional housing	2020
	Woman-led		Resource centers	
	PLE-led		Peer based delivery of supplies	
			Prison ministry	
#2	Black-led	Yes	Laundry	2016
			Family support	
	Woman-led		Care packages	
			Social connection	
			Future goal: transitional housing	
#3	Woman-led	Yes	Mobile navigation	2021
			Hot meals	
			On-site medical care 2x/month	
			Hospitality/Navigation 2x/week	
			• Mailing address	
			• Emergency food bags	
			• Medical access program (MAP) enrollment assistance	
			• SNAP applications,	
			• Document procurement (birth certificates, social security cards, ID cards)	
			• SSI and SSDI applications	
			• IRS filings,	
			• Coordinated housing assessments	
			• Housing move-ins	
			• General case management	
#4	BIPOC and Queer-led	No (in process)	Mobile encampment support: Food, water, harm reduction supplies (fentanyl test kits, Narcan), social services navigation support, laundromat fees	2020
	Grassroots org		Weekly food pantry at community center	
	PLE consultants/advisory board		Robust social media group directly matching community needs with support.	
	Mutual aid group		Warming shelters and hot meals during extreme cold weather	
#5	Church-based	Yes	Food pantry 4 days/week	1978
			Provide bus passes, medication vouchers, clothing	
#6	Black-led	No	Monthly aid distributions (clothing, hygiene supplies, reading materials, shoes, backpacks, blankets, and food)	2009
	Mutual aid group		Transit assistance program (30-day bus pass).	
			Welcome packages for those entering stable housing (cookware, cleaning supplies)	
#7	Black-led, PLE-led	Yes	Youth organizing	2018
			Youth sports and community building	
			Food, water, hygiene products	
			Financial support for families	
			Job counseling for those exiting prison	
#8	Church	Yes	Weekly women’s outreach providing showers, snacks, and clothes.	Church – 1840
			Hot breakfast 2x/week, men’s clothing closet	
#9	Black-owned and Black-led	No	Mobile food pantry	2014
	Healthcare		Connecting neighbors to healthcare and community resources/navigation services.	
	Navigation		Assistance in applying for health insurance	
			Reentry empowerment after incarceration	
#10	Grassroots	No	Weekly pop-up café with hot meals + supplies.	2019
	Mutual aid group		Provides “know your rights” literature and community empowerment to decriminalize homelessness.	
			Trained in mental health first aid	
#11	Black-led, PLE-led, woman-led	Yes	Community building focused on ending generational poverty in women.	2019
			Individual navigation services focused on women’s survival/escaping poverty by assisting with food access, housing, and skill building.	
			Free meal kits	
			Skills training workshops	
#12	LGBTQIA-led, PLE-led, Black led.	Yes	Runs only LGBTQIA shelter in county	2020
			Amplifies QTBIPOC voices	
			Housing navigation and support	
			Fundraising for QTBIPOC tiny home community	
			Mobile outreach/Camp support	
			Peer counseling for QTBIPOC sex workers.	
			Mobile harm reduction supplies	
			Paw Pals- matches recently housed persons with a support animal	
#13	Black-led, PLE-led	Yes	Holistic work program for unhoused neighbors	2022
			Honest pay for the day program	
			Sustained daily work opportunities	
			Apprenticeships (construction)	
			Showers	
			Laundry	
			Clothing	
			Hot meals 2x/day	
			Social support services	
#14	PLE- led	No	Harm reduction supplies	2020
	Woman-led		Drop-in location and mobile outreach	
			Peer-led advocacy and outreach	
#15	Church	Yes	Food	Church est. 1899
			Bus passes	
			Financial support towards bills	
			Addiction recovery resources and support programs	
#16	Church network	Yes	Weekly food pantry	2011
			Childcare services	
			Eviction prevention resources	
			Furniture	
#17	Church network	Yes	Cold weather emergency shelters – food, housing, clothing	1974

*Note*. PLE = people with lived experience; BIPOC = Black, Indigenous, Person of Color.

### Themes

The central theme identified was *boots-on-the-ground.* Two additional themes of *the homelessness response system is inequitable* and *cautious collaboration* encompass participants’ perceptions of their organizational values and beliefs and the ways in which their organizations interface with one another and with the formal HRS.

#### Boots-on-the-Ground

A boots-on-the-ground ethos whereby participants believed their most important role to be that of direct service provider in the community with PEH permeated the overall findings of this study. Within the study sample, there was a range of characteristics with some being entirely volunteer-based and others having a combination of paid staff and volunteer positions. Similarly, while some organizations operated out of a brick-and-mortar location (oftentimes a church), others had no permanent location beyond someone’s garage or extra bedroom used to store supplies. Still others were mobile, using personal vehicles or organization-owned trucks to reach their clients in the community. Regardless of the specific characteristics, however, participants firmly believed that building relationships and connecting with PEH was the most effective way to help the homeless population: *I personally don’t feel like I could say that we are—we help our homeless population if we don’t go out to the encampments and at least have some type of physical contact.* Within this theme, categories include *services & support; mission, not margins;* and *critical emergency response partners.*

##### Services & Support

Participants articulated the services that their organizations provided and described their perceptions of the impact of those services as well as how their services differed from those offered by more formal homeless services providers. The majority of organizations offered services that met survival and instrumental needs of their clients such as food, water, clothing, harm reduction supplies, hygiene supplies, and bus passes. Many also assisted in making and keeping health care and social services appointments. Participants uniformly believed that their organizations were more thoughtful and intentional about these services than are most other homeless services providers. Participants described tailoring their services to the needs of individual clients or of larger encampments that were part of their outreach efforts. For example, one participant described their organization’s monthly community meetings with the unhoused individuals they worked with to get a better understanding of what was needed and what was working well in terms of their organization’s services. They described being asked for a resource guide and their process of developing it and refining it in partnership with the community:

And so we just went through and kind of, like, pulled from a bunch of different sources and then had, like, a month where I would bring a couple drafts to the park and talk to people and be like, “Are these things, like, still around? Can you vouch for these things?” Some people would even say, like, actually, “I don’t like that place.”

Importantly, nearly all the participants perceived that in addition to meeting clients’ basic needs, offering these services provided opportunities to build relationships. One participant detailed the various services that her organization provides to PEH and referred to their approach as “*concierge homeless services*.” She said, *I don't feel like it's hand-holding or babying people, but it's giving people the care that they need to really accomplish steps and get things done. It takes more time to do things that way, but I think people feel like we really invest in them, we really care for them.* Another participant emphasized this relational approach when she contrasted her organization’s approach with that of larger organizations: *When you go to those facilities, you are a number. You are a number and an appointment. So, it’s just like we have our hours that we provide the service…but we’re not going to turn you away because of some silly rules that these other organizations [have]*.

##### Mission, not Margins

This theme captures how participants were motivated by their organizational missions and personal values, sometimes at the expense of their organization’s financial stability and growth. Many organizations were motivated by their faith traditions while others were explicitly secular. Those participants who had themselves experienced homelessness were forthcoming about how their lived experience animated their work. They also shared how the informal nature of their organizations allowed them to live out these values and assumed that public funding entailed burdensome reporting requirements that would necessitate trade-offs: *the more funding that you receive, the more reports that you have to do. And so—it’s almost like the work is not the same, you know.* While participants recognized that their organizations operated on very thin financial margins, they were not willing to give up the flexibility that enabled them to meet their missions. One participant put it most clearly when he said, *“I’m not sacrificing nothing. Not for the dollar because it doesn’t exist in my community right now. We’ve always provided our own resources.”*

Participants also shared how the small, informal nature of their organizations allowed them to more fully live into their mission. For example, participants commonly used their own vehicles to deliver services. One participant shared how she would pick clients up in her personal vehicle as early as 5:15am in order to get them to the methadone clinic by 6:00am and another reported that she and her small team regularly drive people to various appointments. Yet another described her organization’s efforts during an extreme cold weather event: *So, we had volunteers driving around, looking for unhoused people who were on the streets in the cold with no resources -- no camps, no tents, no, you know.* Participants recognized that this flexibility was largely due to the fact that they did not have much external accountability: *we don’t want to have, like, any kind of funding where there’s strings attached or things we have to do or things we can’t provide. Yeah, we kind of just, like, let the community guide what they want us to do versus, you know, funders or anything like that.* Participants emphasized their accountability to those receiving services (PEH) versus an external funder as a key difference of being an informal organization.

##### Critical Emergency Response Partners

Participants described the critical roles their organizations had played during recent emergencies. They recounted the early days of the COVID-19 pandemic when nearly all social services agencies shifted to remote work. One participant from a small mutual aid organization characterized that time thusly:

And that April of 2020, when no other group was out there, nobody was out there, it was just us and that small Baptist group that was giving out soup right around the corner, we got a lot of tears that time around because a lot of the services and the providers that they depend on dried up overnight. Just went away.

Participants lamented the lack of response from traditional homeless services and described both how the needs of PEH changed during the pandemic as well as how their organizations shifted in order to meet those needs. For example,

Clothing was hard. Clothing closets were not open. And we actually opened a…clothing closet for men…probably starting in… summer of ‘20…because we did not have a clothing closet available for the breakfasts. That had not been a part of that program, so we started that and were able to, to run that and provide like a shirt, a pair of pants. But for some of the neighbors that would come to the breakfast, they were not able to access clothing anywhere else, and they were not able to access laundry. So we were that for them.

Participants also described their organizational responses to Winter Storm Uri, a winter event in Texas that left 40% of the community without power and many without water during below-freezing temperatures for several days in February 2021. One organization facilitated a campaign to get safe water to the community, and several described picking individual PEH up off the street and driving them to shelter. Common to all these efforts was the lack of communication from formal emergency response partners and therefore lack of coordination between individual organizational efforts and a city-wide response.

##### The Homelessness Response System (HRS) is Inequitable

The next theme encompassed participants’ beliefs about the HRS and the ways in which these beliefs influenced the work in which their organizations engage. Participants clearly believed that the larger, more formal organizations in the HRS could and should be doing more to support PEH and to prevent and end homelessness, but city politics, bureaucracy, and misaligned metrics contributed to an inequitable system in which money—and, therefore, power—is directed to larger organizations thereby excluding smaller, often Black-led groups from needed resources. Within this theme, the categories are *needs not being met* and *larger organizations should share.*

##### Needs not Being Met

Participants articulated their beliefs regarding how the exclusion of smaller, informal groups from the formal HRS resulted in the needs of Black PEH not being adequately met. Specifically, Black participants and participants with lived experience of homelessness cited a lack of trust in formal institutions on the part of PEH that could interfere with their willingness to seek out or accept services:

So, for a Black person out there on the streets that's experiencing homelessness, they might not have the trust from the City or from other entities out there, so they might—even though they need them, they might deny them because “I don’t trust you.” You know, “You always failed me. You are part of the reason that I’m in this situation whether you admit it or not.”

Participants also perceived that the services and support provided by larger organizations were not as culturally appropriate as the care provided by their smaller organizations. One participant recounted a specific example:

I had a lady come up to me because, you know, we were both African American. And she came up to me and said, do you have some hair grease? And I was like, I sure do. Because this hair takes grease…If you’re outside of the African American community, that’s not something that’s on your mind in that way. And we started providing grease out there…And nobody was doing that before.

##### Larger Organizations Should Share

Participants believed that larger organizations within the HRS were awarded funding despite not using their resources to their fullest potential. Participants also articulated how their lack of organizational infrastructure often precluded them from accessing public funding dedicated to homeless services. One participant said:

I mean, it might be 20 pages to fill out a grant request, but then, it’s data that I don’t have maybe because I’m a new organization or questions I don’t even understand what they’re asking for. And, I’m, I’m a pretty smart person, and I can write well enough to, to apply for a grant. But then, it might say… attach your racial discrimination policy handbook or whatever, where I would have to create a handbook to even attach it to answer a question. It's just a lot of barriers to even accessing that money.

Participants acknowledged the limited resources available in the community and did not suggest that their small organizations should assume the roles of the larger organizations within the HRS. They did, however, voice frustration that larger organizations were not boots on the ground and were therefore not aware of nor adequately meeting community needs. They also wished that larger organizations acknowledged the important work of informal groups by partnering to share resources. Participants perceived growing needs and expressed a desire for more cooperation amongst homeless services organizations:

it just seems like there needs to be…a great increase in terms of cooperation and working together because, you know, like I said before, our food pantry is busier now by double than ever before, and it’s not going to change anytime soon.

Another participant suggested formalizing partnership requirements:

when people are seeking funding, that—the question always pops up in an application. It says, “Do you collaborate with other organizations?” …can we change that and say, “What is the organization that you are partnering with?” So, like, always make it where two or more organizations are applying for this funding.

#### Cautious Collaboration

The next theme, *cautious collaboration,* encompasses how informal organizations collaborate with other informal organizations, with larger homeless services organizations, and with health and social services providers to fill gaps in services. Within this theme, categories include *ad hoc* *approach* and *communication channels.*

##### Ad hoc Approach

Participants recognized substantial need for medical care and described a variety of approaches to connecting clients to formal health or mental health resources. No one, however, recounted a systematic way to ensure their clients were receiving appropriate medical care for mental or physical health needs. This was commonly due to a lack of awareness of available resources. For example, some participants reported calling 911 when clients were in crisis and others indicated that that would be the solution should a need for services arise. One participant said, “*We immediately called 911, and it was, it was a mental issue… You know, there's, there's nothing we can do for someone in that state, you know.”* The ad hoc approach to service connection at times stemmed from participants’ own lack of trust in formal institutions. One participant described her work thus, *“My role is kind of filling the gaps in the services that exist, like connecting people with the resources. I feel like a lot of our work is just fighting against the, the formal, like, systems in place and advocating for people.”*

Participants described efforts to remove barriers to care for their clients such as providing transportation to appointments and providing information for local healthcare providers. A few participants mentioned that their organizations had incorporated questions regarding health insurance status and health needs into their intake process in order to better understand the needs of their clients. Participants also perceived, however, that their referrals and advocacy on the part of their clients sometimes went unheeded. One participant said, *“support these smaller organizations in having a voice, having more opportunity, and being listened to. You know, like, when we say, ‘We found this person who's really vulnerable and this person needs to be prioritized.’”*

##### Communication channels

The final category within this theme articulates the ways that information filtered through the community as well as how participants accessed resources and identified individuals and organizations with whom they could partner. Participants described their boots-on-the-ground approach to networking and relationship building which they believed to be fundamental to their access to information. They also emphasized their connections to individuals and not necessarily to organizations. A handful of individuals at well-established homeless services organizations and within local law enforcement were named by several participants as being important purveyors of information and facilitated the ability of the smaller organizations to access resources. One participant described it thus:

So I, I would actually say that we don’t interface with organizations. We interface with people inside of organizations. So we don’t have any like actual, like, you know, like signed agreements or anything like that…but there are people within those organizations that we usually actually collaborate with either through text or e-mail.

Participants described frustration in the lack of communication regarding available resources for clients, how to access those resources, and who was eligible for them. They also desired to be part of the solution of connecting the individuals with whom they worked to the resources that were available. One participant said, *“I wish I knew more about what organizations were doing and if people are connected and, and how to better… support the work that… some of you all [in reference to other focus group participants] are doing and how can we be better at that.”* Another described the approach that his organization had taken to facilitate communication:

we started a monthly, citywide outreach meeting for all the outreach teams here…We generally meet like once a month, and, like, we’re focusing on, like—you know, we’ll have a goal. To me and [organization], that’s, like, very important, especially with the landscape it is now, turnover rates at organizations…It’s to see what everybody is doing, just to collaborate, because again, I, I do believe there’s like not one organization that can do everything for someone.

## Discussion

In this study, we focused on how informal organizations that provide services and support to PEH operate and sought to identify if there are disparities in efficacy or access to these organizations. We used fundamental cause theory as the overarching theoretical framework to illuminate how the organizations identified by our key informants promote access to health-promoting resources including knowledge and beneficial social connections among minoritized and marginalized PEH. Our findings reveal that the relational approach employed by our participants helped to bridge the chasm between the knowledge, skills, and ability of PEH and their needs for food, clothing, hygiene, and shelter; emotional support and hope; and assistance navigating complex healthcare, housing, and social systems. Thus, their boots-on-the-ground approach allowed informal organizations to reach many PEH who are unable or unwilling to access services through more established pathways, thereby overcoming some of the barriers faced by stigmatized PEH. Our findings suggest that informal groups may be successful in reaching stigmatized PEH because of their approach and focus on building community and sharing knowledge and resources among and between PEH and themselves as providers.

The utility of this approach was particularly evident during the earliest phases of the COVID-19 pandemic as the established relationships with PEH allowed informal organizations to understand and quickly shift services to meet changing needs in the community. This is similar to prior research indicating that peer support models are successful in meeting the needs of marginalized individuals in large part because peers fill gaps in care created when traditional systems fail to respond to community needs in culturally-appropriate ways.^40^ Future research should investigate the extent to which critical components of individual-level peer support such as mutual understanding, empathy, and support^41^ operate similarly among community-level peer support such as that provided by informal groups like those represented in the current study. A better understanding of the critical components of informal groups is needed as this would help to ensure that any capacity-building efforts or efforts to expand services would not inadvertently diminish those core characteristics. Future research should also explore ways for incorporating smaller organizations into formal public health preparedness and response efforts to more effectively and efficiently reach marginalized PEH.

We also employed social network theory to explore relationships among informal organizations and to identify how, when, and why these groups collaborated with the formal homelessness response system. Exploring these relationships identified the importance of informal social networks amongst and between individuals within informal and formal homeless services organizations. Our findings suggest well-connected individuals within the larger homelessness response ecosystem serve as conduits of information and resources to informal organizations but do so in an ad hoc manner that requires motivated individuals on both sides of the communication channel. This echoes prior research investigating interorganizational networks and how those networks can help strengthen community partnerships and improve service delivery.^42^ However, while these informal communication channels allow information and some resources to be shared, the high rates of staff turnover within the homeless services sector^43^ presents challenges to ongoing communication. Further, the reliance on individuals within organizations to filter communication also presents challenges to transparency due to lack of accountability for sharing information or resources. This is problematic because it can result in informal organizations being unaware of important community-level resources and therefore unable to connect their clients with these services, which was reflected in our study’s findings. To the extent that informal organizations are preferred locations for Black PEH, a lack of communication infrastructure may also exacerbate racial inequities due to clients of these organizations not being referred to needed resources.

Reliance on informal communication channels can also contribute to already lengthy wait times for services which have been found to be substantial barriers to service utilization in homeless populations.^44,45^ Informal communication channels and lengthy waits for services may also disproportionately impact those with high service needs as they contribute to needs going unmet and reinforce convoluted referral mechanisms.^46^ A study examining the use of a listserv as an informal communication and collaboration tool among homeless service providers demonstrated the importance of informal low barrier communication but also the need for improved integration of systems for referrals and access to resources.^47^ Future research should explore low-barrier solutions for facilitating more formal communication and referral mechanisms that can incorporate the diversity of provider organizations within a local community.

Findings from the current study also reinforce how difficult it is to apply for and obtain government funding for providing outreach and engagement services for PEH. Currently, most of the funding for homeless response efforts is funneled from HUD through CoCs to local nonprofit organizations and government entities. HUD funding is notoriously restrictive in how funding can be allocated and on which types of services their funding can support. For example, program types funded by HUD typically focus on housing, and local policies do not prioritize “supportive services only” programs with which smaller organization service models more closely align. More broadly, the administrative burdens that accompany 501c3 non-profit status can quickly swamp the capacity of small organizations to continue their services, meet their missions, and also attain compliance with accountability standards.^48^ Our findings similarly identified that informal organizations prioritize being in the community with PEH and recognize that they would likely have to sacrifice already scarce human resources to be able to competitively apply for funding. Taken together, these findings suggest that the current funding structure may be contributing to ongoing systemic inequities that plague many homeless response systems (NAEH, 2021)^49^ in that many informal organizations are Black-led and/or led by people with lived experience of homelessness. This finding warrants further investigation to clarify how traditional funding streams may reinforce systemic inequities and if incorporating non-traditional CBOs into the formal HRS can help to rectify racial disparities by re-distributing funding, resources, and decision-making power in ways that allow both smaller organizations and the people they serve to access such. To gain a more comprehensive picture of the homeless services system landscape, future research should also quantify the proportion of organizations within local communities that are Black-led and/or led by people with lived experience of homelessness and examine the extent to which they are included within the formal HRS.

Our results also clarify the difficulties facing these largely volunteer-based groups that typically work outside of the formal HRS and clearly point to how they remain disconnected from having a role in the vision, strategic planning, and priority-setting of the larger homeless services ecosystem. This is in line with prior research that identified the need for integration of homeless service systems planning, implementation, and evaluation at all levels.^50^ A possible solution to incorporating smaller organizations and building a more integrated and equitable HRS may be community care hubs. Community care hubs (CCHs) are typically led by a community-focused institution that collaborates with and supports several CBOs to address health-related social needs. Evidence indicates that they are an equitable solution to an integrated system because small CBOs have a seat at the table with larger health delivery organizations, all of which have an equal voice and the goals of all organizations can be incorporated into decisions about resource allocation, funding, and overarching goals.^51^ A CCH could be a solution that allows smaller organizations to have a voice and more financial stability in a formalized system while maintaining their client-driven mission. Further, CCHs could rectify the failure of the formal HRS to recognize informal organizations’ expertise and intimate knowledge of the hyperlocal issues that impact their communities and extend the capacity of these groups to raise awareness of increasing or changing needs of the communities they serve. Future research should explore the potential to implement CCHs and identify the translational facilitators and barriers to this solution.

### Limitations

The study’s small sample size and its setting reflect conditions specific to the participants and local community and are not generalizable to all informal organizations across the U.S. However, we have addressed transferability by providing detailed descriptions of the context and by using direct quotations. Further, the data collected reflect the perspective and opinions of organizational representatives and may overlook other key organizational characteristics. Future research could use the emerging evidence of the current study and employ a case study approach to more closely examine relevant organizational characteristics. Future research could also use standardized measures that assess organizational characteristics to complement and extend the current study’s findings. It can be seen as a limitation that the researchers did not verify interpretations with participants, but emerging findings were checked with key informants and their feedback was incorporated. The key informants provided critical insight in all stages of this research project, but the small number of informants (*n =* 7) limits the representativeness of their input and the subsequent study findings.

## Conclusions

The findings of this study raise important questions about the current structure of homelessness services and the limited integration of informal service providers. Informal and non-traditional homeless services providers including mutual aid groups and faith-based organizations are critical partners in homelessness response and are often preferred by Black PEH. However, these smaller groups remain disconnected from the larger HRS ecosystem which underscores the importance of better integration of services as well as a needed reconceptualization of how funding is allocated in order to improve equity, reduce system fragmentation, and alleviate frustration on the part of both PEH and informal organizations. Efforts to incorporate informal organizations into the formal HRS or to build capacity of these organizations must be done thoughtfully and be led by informal groups themselves alongside people with lived experience of homelessness. Without careful attention to the expectations and assumptions of formal social services, informal groups would likely lose their ability to remain flexible in the face of shifting needs which would likely undermine relationships and connections to racially minoritized and marginalized PEH.

## Supplemental Material

Supplemental Material - Racially Equitable Homeless Services: Exploring Organizational Characteristics Supplemental Material for Racially Equitable Homeless Services: Exploring Organizational Characteristics by Whitney Thurman, Elizabeth Heitkemper, Tara Hutson, Summer Wright, Amy Patten, and Andrea Kaltz in Community Health Equity Research & Policy.
