# Supplementary material for: Racially Equitable Homeless Services: Exploring Organizational Characteristics
Source: Community Health Equity Res Policy. 2025 Feb 20;46(3):231–43. doi: 10.1177/2752535X251321535 (PMC12963461; doi:10.1177/2752535X251321535)
Supplement: Supplemental Material - Racially Equitable Homeless Services: Exploring Organizational Characteristics [file sj-pdf-1-qch-10.1177_2752535X251321535.pdf]

Individual Interview Guide

1. Please describe your role at your organization.
    a. For how long have you been in this role? Is this a paid position? What do you enjoy about this role?
    b. For how long have you been involved in homeless services?
    c. How does this role compare to other jobs in homeless services that you may have had or that you know about?

2. Please describe the history and work of your organization.
    a. Can you tell me how long has your organization been providing services to unhoused populations in Austin/Travis County?
        i. What types of services/care do you provide? How have these changed over time?
        ii. How do the people you serve/care for find you/come to know about your services?
    b. Can you describe why [your organization became involved in this work?
    c. Please describe the overall structure your organization.
        i. How many employees/volunteers?
        ii. Types of roles
        iii. Funding sources
    d. Can you share a little about your organization's mission, vision, and values?
        i. How do you think these values are enacted in the work you do in providing care and support for unsheltered Black adults?

3. In your opinion, how does the support/care provided by your organization compare/contrast with services that unsheltered adults may receive through formal health or social services systems (e.g., X, X)?
    a. Why do you think unsheltered Black adults might prefer or seek out the services/care provided by your organization?
    b. How does the care and support that your organization provides benefit unsheltered Black adults that is unique from services in the formal system?

4. Please describe how your organization's work shifted during the early phases of the pandemic (i.e., March – August 2020)? How did your response evolve as the pandemic continued?
    a. Did your clients/care recipients have different needs?
    b. Did the population change?
    c. Did the locations in which you work change?

5. In your opinion, how did homeless services shift in response to the pandemic?
    a. Did they shift? Were the shifts beneficial?
    b. What would you keep moving forward?

6. If you had a magic wand, what is one thing that you would change about the homelessness response system in Austin/Travis County in order to be more responsive and more inclusive of the needs of diverse groups?

7. Is there anything else you would like to add that I didn't ask?

Note: The following probing questions will be used, as appropriate, throughout the interview:

Would you give me an example?
Can you elaborate on that idea?
Would you explain that further?
I'm not sure I understand what you're saying.
Is there anything else?

Focus Group Guide

1. How does your organization interface or interact with other homeless services providers?
    - How do you communicate with other organizations?
    - Do you receive referrals from other organizations? If so, how? How often? For what?

2. How does an individual typically become involved with your organization to receive services?
    - Probes: Referred? Word of mouth? Partnerships with other organizations? Street/direct outreach?

3. How does your organization connect individuals to medical or mental health services?
    - Is there a standard process? What is it?
    - When do you make the connection?
        - When a client requests it?
        - When you or a person from your org thinks a person is in need?
    - What would make this process easier?

4. The ability to easily share information between organizations has been identified as very important to this work.
    - How do share client information? With whom do you share it?
    - Do you have need to track client information?

5. To what extent does your organization interface or interact with [name of lead agency of local CoC]?
    - For what reasons do you work with them?
    - What is your understanding of the CoC's role?
    - What do you wish they would or could do?

6. To what extent does your organization work with the City or County?
    - For what reasons do you work with them?
    - What is your understanding of their role?
    - Do you seek information or help from either of these entities?
    - What do you wish they would or could do?

7. Are there other organizations or entities that you look to for guidance or funding opportunities?
    - If so, what are they?
    - How did you come to know about the organizations or the funding opportunities?
    - Are there organizations to whom you look for mentorship or assistance with your work? How did this relationship get established?

8. Generally, how do you become aware of resources that might help your organization in carrying out its mission?
    - Once you are aware of resources, do you share them with other organizations? If so, how?

9. Can you provide an example of a partnership or collaboration that you have that you think works very well?

- What makes it work well?
- What would you change or improve if you had the capacity and resources?
    - What capacity and resources would you need to make these changes?

10. What do you think could help the provider community more easily partner and innovate?

- What would be most helpful to you?

12. An important theme from the interviews was flexibility, which was often discussed within the context of funding and capacity. Many participants acknowledged that without more funding, they were limited in the work they could do. But they also acknowledged the burdensome strings associated with many funding sources. That is, city or state funding might prevent some services while requiring others that are less central to your mission.

- So, keeping this in mind, what aspects of your work or mission would you not sacrifice for more funding?
- Are there areas of your work that you would consider doing differently or not doing in order to receive additional funding?

13. How does your organization evaluate success?

- How do you know if you're meeting your mission?
- What does your organization need to be better able to meet its mission?

14. What would you like to add that we have not covered?
